# The Moral Importance of Reflective Empathy

**DOI:** 10.1007/s12152-017-9350-7

**Published:** 2017-12-15

**Authors:** Ingmar Persson, Julian Savulescu

**Affiliations:** 1 Department of Philosophy, Linguistics, and the Theory of Science, Göteborgs universitet, Gothenburg, Sweden; 2 Oxford Uehiro Centre for Practical Ethics, University of Oxford, Suite 8, Littlegate House, 16/17 St Ebbe’s St, Oxford, OX1 1PT UK

**Keywords:** Altruism, Paul Bloom, Empathy, Sense of justice or fairness, Morality, Jesse Prinz

## Abstract

This is a reply to Jesse Prinz and Paul Bloom’s skepticism about the moral importance of empathy. It concedes that empathy is spontaneously biased to individuals who are spatio-temporally close, as well as discriminatory in other ways, and incapable of accommodating large numbers of individuals. But it is argued that we could partly correct these shortcomings of empathy by a guidance of reason because empathy for others consists in imagining what they feel, and, importantly, such acts of imagination can be *voluntary* – and, thus, under the influence of reflection – as well as *automatic*. Since empathizing with others motivates concern for their welfare, a reflectively justified empathy will lead to a likewise justified altruistic concern. In addition, we argue that such concern supports another central moral attitude, namely a sense of justice or fairness.

## What Empathy Is and the Case Against its Moral Importance

It has generally been taken for granted that empathy is important for morality, so it was to be expected that this assumption would eventually be challenged. Paul Bloom’s *Against Empathy* [1] is a full-scale challenge; Jesse Prinz makes a similar, smaller scale challenge in *Is Empathy Necessary for Morality* [2] and *Against Empathy* [3]. We will argue that empathy can have an essential role to play in moral motivation, but then it needs to be harshly disciplined by other factors – in particular, reasoning – to play its role properly.

To begin with, what is empathy? Bloom understands it to be ‘the act of feeling what you believe other people feel – experiencing what they experience’ [1, p.3]. Prinz’ conception is similar: ‘it’s feeling what one takes another person to be feeling’ [2, p.212]. Bloom distinguishes empathy from cognitive empathy [1, p.17], sympathy and pity [1, p.40], and compassion and concern [1, pp.40–1]. For instance, ‘if I understand that you are feeling pain without feeling it myself’ [1, p.17], this is cognitive empathy. Cognitive empathy is ‘morally neutral’ [1, p.38]: people who are morally good share it with ‘successful con men, seducers, and torturers’ [1, p.37]. So, Bloom seems to think – correctly, to our minds – that cognitive empathy is not enough to motivate moral behaviour, whereas empathy in the proper sense – ‘emotional empathy’ – can be. Indeed, what he sees as defects of empathy implies that it is a motivator. He objects that:

it is a spotlight focusing on certain people in the here and now. This makes us care more about them, but it leaves us insensitive to the long-term consequences of our acts and blind as well to the suffering of those we do not or cannot empathize with. Empathy is biased, pushing us in the direction of parochialism and racism. It is shortsighted, motivating actions that might make things better in the short term but lead to tragic results in the future. It is innumerate, favoring the one over the many. It can spark violence; our empathy for those close to us is a powerful force for war and atrocity toward others. [1, p.9].

These critical remarks imply that empathy motivates; Bloom’s point is that empathy often motivates us in ways that are morally objectionable. Thus, ‘empathy is not sufficient to guide moral action’ [1, p.191], but it is not disputed that it is a motivating factor, that it could make us ‘care’ about others.

Empathy, then, is not a reliable guide to moral action because it is directed first and foremost at people (bracketing other sentient beings for present purposes) we know well or who are present before our eyes – those who are ‘spatially near’ – at the expense of those who are strangers to us, or beyond the reach of our senses. With respect to people who are spatially near – including ourselves – it is especially focussed on how they will fare in the more immediate future. That is, its focus is on what is temporally near as well as spatially near. Furthermore, even among people who are present to our senses, some may not easily be the target of our empathy because they are different from us in some conspicuous ways: their skin colour is different, they are deformed, dirty, etc. – that is, empathy is discriminatory. Finally, empathy is ‘innumerate’: we cannot empathize with groups of people in proportion to their number. The larger the group, the more of a drawback this limitation is.

Prinz voices similar objections to empathy: it ‘may lead to preferential treatment’, ‘may be subject to unfortunate biases, including the cuteness effects’, ‘is prone to in-group biases’,‘is subject to proximity effects’, and so on [2, p.226]; cf. [3, pp.227–30]. Yet he adds to the list of accusations that ‘empathy is not very motivating’ [2, p.225]. But this is hard to square with what he says to support his other accusations. As regards preferential treatment, he reports that when subjects had been presented with a vignette about a woman awaiting medical treatment, ‘they overwhelmingly elected to move her up at the expense of those in greater need’ [2, p.226]; cf. [3, p.228] who were anonymous to them. And as regards in-group biases, he refers to some studies that have ‘found that empathy leads to helping only when the person in need is a member of the in-group’ [2, p.226]. But then empathy does after all motivate us to favour or help some. Thus, the more plausible moral objection to empathy is not that it is not motivating, but that it often leads us morally astray because it *is* motivating.

Bloom does not have that much to say much about the attitudes of sympathy, pity, compassion and concern to which he is more favourably disposed than empathy (as Prinz is to concern, [3, pp.230–1]). He writes that ‘*sympathy* and *pity* are about your reaction to the feelings of others, not the mirroring of them… If you feel bad for someone in pain, that’s sympathy, but if you feel their pain, that’s empathy’ [1, p.40]. We believe the same holds for compassion: it is the emotion of feeling sad or unhappy because you believe that someone else is suffering or is having a hard time. By contrast, we regard being concerned about others, or their welfare, as a desire that things go well for them for their own sake. To be concerned about someone in this sense is to adopt an attitude of benevolence or altruism towards them. If you have the power to see to it that things go well for those towards whom you have adopted this attitude, you need not be put in a position in which it is proper to feel sympathy, pity or compassion for them, that is, to feel bad or sad because things are *not* going well for them.

Bloom insists that concern and compassion do not require empathy [1, p.41]. This allegedly makes them ‘more diffuse than empathy’; as a result, it is ‘weird to talk about having empathy for the millions of victims of malaria, say, but perfectly normal to say that you are concerned about them or feel compassion for them’ [1, pp.40–1]. Thus, on his view, concern and compassion are not innumerate as is empathy, and exposed to criticism on this score. Due to the fact that compassion, like pity and sympathy, are in place only if the subjects they target are faring badly in some way, we will concentrate on the attitude of benevolent or altruistic concern and ask what motivational role empathy does or could play in relation to this attitude, that is, for a desire that others fare well for their own sake – though we may in fact empathize more frequently with others when in fact they do not fare well.^1^

But more needs to be said about what empathy is – or rather what it will here be taken to be. It is not actually *feeling* what you believe others to be feeling, as Bloom and Prinz would have it. For instance, when you empathize with somebody whom you believe to be feeling physical pain, e.g. because they have hit their thumb with a hammer, you do not feel physical pain; instead, you more or less vividly *imagine* feeling a pain like the one you believe they are feeling. You imagine what it is like to be them, feeling what they do. Notice that it is not imagining that *you yourself* are feeling what you believe they are feeling (which is what e.g. Smith usually takes sympathy to involve); it is imagining *being them*, feeling as they are believed to be feeling.^2^ However, it would be too strict to demand that empathizing with someone requires succeeding in imagining feeling something which is quite similar to what this individual is *in fact* feeling. You may be said to empathize with someone when you imagine feeling as you believe they do, though your belief is only very roughly right. Nonetheless, empathizing requires imagining having the right *kind* of feeling: for instance, you cannot be said to be empathizing with somebody if you imagine being glad when that individual is in fact sad.

According to this conception of empathy, it is a mistake to talk about *feeling* empathy, *emotional* empathy, or empathic *feelings*, as Bloom and Prinz do. This is a respect in which empathizing with someone’s feelings differs from what is often called *emotional contagion*: for example, if you are surrounded by sad people, this is likely to make you sad, while if you are met with smiles, this liable to put you in a good mood. In these cases, you are actually feeling the emotions that others are having, not imagining feeling them. Bloom notes that emotional contagion is ‘not quite the same as’ empathy [1, p.40], but he fails to see this salient difference. Prinz’s claim is more explicitly inconsistent with the present understanding of ‘empathy: ‘empathy in its simplest form is just emotional contagion’ [2, p.212].

Anyway, empathizing with somebody, as we conceive it, is imagining feeling how this individual is feeling, especially in ways that are good or bad for him or her.^3^ Admittedly, the term ‘empathy’ is often used in other ways, and this accounts for the fact that it may seem odd to say, e.g. that *Schadenfreude* or malice involves empathy. But to our minds, there are other terms, like ‘sympathy’, which are better suited to designate what is at issue here (or a technical term like ‘emotional empathy’ may be introduced).

Some of Bloom’s arguments against empathy trade on the failure to separate it clearly from emotional contagion. He correctly points out that we might find ourselves sad without realizing that this is the result of the sadness of others having infected us. Then we will not be motivated to do anything to relieve their sadness: ‘Without an appreciation of the source of one’s suffering, the shared feeling is inert’ [1, p.173]. But we cannot intelligibly empathize with someone’s sadness without realizing that the sadness we imagine feeling is the sort of sadness that we believe this individual to be feeling because it is imagining feeling a sort of sadness precisely for the reason that it is the sort we believe this individual to be feeling. Thus, empathy cannot be motivationally inert because it lacks a target in the sense described by Bloom.

At some points, Bloom’s misconception of empathy as comprising actual feelings appears to mislead him to overlook the involvement of empathy in concern. He writes: ‘you see the victim’s face contorted in anguish, but you don’t see anguish in the consolers, just concern’ [1, p.175]. However, in so far as the consolers empathize they do not *feel* anguish, but *imagine* feeling it, and it should not be expected that imagined anguish needs to show up in the face in the same fashion as genuine anguish does. If this imagined feeling motivates concern, it is precisely concern that we should expect to see in the face of an empathizer, not anguish.

## Spontaneous Empathy and Voluntary, Reflective Empathy

We have seen that cognitive empathy is not sufficient to make us concerned: if we acquire a belief that somebody is suffering on the basis of a verbal report, we might not be the least motivated to relieve the suffering. On the other hand, empathy with sufferers does motivate us to relieve the suffering. It has already been observed that if that were not so, Prinz and Bloom’s case against empathy to the effect that it is a poor guide to moral action, since it is biased and innumerate, would be undercut. So, let us proceed on the plausible assumption that it does motivate.^4^

It is true that, as Bloom maintains, we ‘cannot empathize with more than one or two people at the same time’ [1, p.33]; cf. [2, p.229]. It also true that we find it harder to empathize with people who are different from us in conspicuous ways, such as having a different skin colour, or who repel us by being deformed, dirty, etc., and that this is likely to lead to discrimination, or exclusion of individuals from concern on morally unjustifiable grounds. Empathy is also biased towards the near future in the sense that we empathize more readily with the suffering that individuals – ourselves included – will feel in the near than in the more distant future. These are reasons why Bloom – rightly – thinks that ‘empathy is a terrible guide to moral judgment’ [1, p.45].

On the other hand, he admits that ‘it can be strategically used to motivate people to do good things’ [1, p.45]. (In this respect, he appears more conciliatory than Prinz, as will be seen below.) The question then arises whether it is not a better strategy to try to discipline empathy than try to do without it. The latter seems to be what Bloom recommends when he claims that ‘on balance, we are better off without it’ [1, p.39]; similarly, Prinz hopes for ‘the extirpation of empathy’ [3, p.228]. This is primarily where we part company. The risk is that without empathy we would not be concerned with anyone’s well-being, not even our own beyond the present moment. As has been seen, Bloom believes that such concern does not require empathy, but he concedes that cognitive empathy is not sufficient for concern when he writes that people who lack concern can have it, e.g. con men, torturers, and psychopaths. What, then, could fill the slack left by cognitive empathy? Bloom does not seem to answer this question.

His appeal to compassion and concern are unhelpful because they are biased and innumerate just like empathy: we feel more compassion and concern for suffering that is close in time, for people who we know well and who have been friendly towards us, and for single identifiable individuals than for masses of anonymous people. A readily available explanation of this fact is that empathy is the motivational source of these attitudes.

Empathy with someone is capable of motivating because it is imagining what it is like for this individual to have a (positive or negative) sensory or affective experience, and this consists in having an ‘image’ or, better, a sensuous representation of the experience which is similar or isomorphic to the experience and derived from having this kind of experience oneself. Thus, as Bloom notes [1, pp.147–9], in order to be able to empathize adequately with somebody who is feeling pain, say, it is necessary to have felt a similar pain yourself – so, those rare individuals who are congenitally insensitive to pain cannot empathize with those who are exposed to pain. Since having pain motivates you to try to rid yourself of it, it would not be surprising if imagining somebody having the pain that you believe this individual to have could motivate you to try to rid them of it, given that the imagined pain is similar to an actually felt pain. The evolutionary explanation of why we so readily imagine feeling a pain we think we will ourselves suffer if we do not take action is surely that this will motivate us to take action to avoid the pain. Then, provided it could serve our reproductive fitness, we should expect that the same device is put to use in the case of others, so that imagining someone else having a pain could also motivate action to save them from the pain, as both commonsensical experience and experimental evidence indicate. But since the imagined pain is not as vivid or forceful as a felt pain (unless it is hallucinatory), it will not motivate to the same degree (cf. [4, p.764]).

If you had been hooked up to someone else’s nervous system by something functioning like afferent pathways, so you actually felt the pain this individual feels in his or her body, you would be more strongly motivated to eliminate it, probably as strongly motivated as this individual. But then you could not *empathize* with the pain this individual is feeling for the same reason that you cannot empathize with the pain that you are now feeling in your own body. The reason is that you cannot *imagine* feeling a pain that you are actually feeling; the actual feeling so to speak overshadows any imagined feeling. It follows from this that Prinz is right when he points out [2, p.214]; [3, p.219] that empathy cannot be necessary for each and every moral attitude that we could adopt, e.g. the indignation that we may feel because of the pain we are now suffering unjustly.

When you attribute the pain you imagine to someone else, it is their pain you will seek to relieve in the first instance. At a pre-linguistic stage, the belief that another is in pain would have to be expressed in a medium of sensuous representation; so there would then be no distinction between so-called cognitive empathy and empathy in our terminology. You would be moved to some extent to relieve the pain you imagine the other to be having. But if you cannot relieve the pain of the other, you may resort to relieving only the pain you imagine and, thereby, the pity you feel. As Bloom mentions [1, pp.74–5], if you empathize with the pain of somebody writhing in front of your eyes, and find that you can do nothing to remove the pain of this individual, you may resort to changing your location so that this individual is no longer within sight. For if you can no longer see this individual, your empathy is liable to subside and, thereby, your desire that the victim’s pain be relieved, alongside the pity and frustration that you will feel because this desire cannot be satisfied.

If the account of empathy given here is correct, is it possible to ‘exploit people’s empathy for good causes’ [1, p.49], by trying to give it better direction? Such a strategy would seem preferable to the strategy of removing empathy that would risk leaving us motivationally dry and unconcerned about the weal and woe of our future selves and others. According to the account given here, we are capable of modifying the direction of empathy because it is not only true that we *spontaneously or automatically* imagine having sensory experiences we believe that others undergo or that we might undergo in the future; we can also do this *voluntarily or at will*.^5^

To take a simple case involving only yourself: if you are told that you will probably feel acute pain later today, you will immediately be seriously concerned, think desperately about ways of escaping this pain, and feel fear it cannot be avoided. This is because you automatically empathize with yourself later today, i.e. you automatically imagine feeling the pain you believe that you might feel shortly.^6^ By contrast, your automatically imagining the acute pain you hear that you might suffer next year will be much more fleeting and perfunctory, if it occurs at all. But your *reason* could inform you that your pain next year will one day be just as real and unbearable as your pain later today, and this may induce you to imagine *voluntarily* feeling next year’s pain if this could help motivate you to find ways of avoiding it. If this imagining is done with some persistence, it will most likely increase your concern about feeling this pain, though it is unlikely to become as great as your concern about the pain later today, since the outcome of your act of voluntary imagination will probably be less vivid or detailed. True, a voluntary act of imagination presupposes that you are motivated to perform it, but the deliverance of your reason along with your automatic empathy, though perfunctory, may be enough to supply this amount of motivation which could inflate itself by means of a voluntary act of imagination.

Thus, we can counteract our bias towards the near future in matters concerning ourselves, though we are unlikely to overcome it completely. More often than we would like, it will continue to make us act in ways that we recognize as weak-willed and irrational. But although it would *now* often be better for us to be able to rise above this bias, it has probably served us well in the past before the rational powers of our species developed to anything like the present extent because the situations that it is most pressing to deal with are as a rule those in the nearer future.

Similarly, from an evolutionary point of view it is not hard understand why our empathy with others is spontaneously selective. Spontaneously, we empathize with individuals who we know well and with whom we have cooperated advantageously, like our kin and people in our community, and not with strangers who might be treacherous or hostile for all we know. This makes evolutionary sense if benevolent or altruistic concern rides on the back of empathy because then we shall be concerned about people in our own tribe and unconcerned about outsiders, and this is likely to make our own tribe successful in the struggle for resources with these outsiders.

But although we do not spontaneously empathize with some individuals for such reasons and, therefore, have little or no concern for them, our intellect can tell us that some or all of the indicated exclusionary reasons are not sound reasons to think that the suffering of these individuals is morally less bad. Suppose that we are repelled by some people because they are deformed or dirty and therefore do not take time to empathize with their suffering. Then, on reflection, we could realize that these features are not anything for which they are responsible and which makes their suffering morally less important. This gives us reason to make a voluntary effort to imagine vividly the suffering of these individuals, despite their unappealing features. As a result, we will be more concerned about their suffering, though probably not as much concerned as we are about the suffering of those whom we find congenial, and with whom we automatically empathize.

Consequently, by means of our power of reasoning, we can expand the range of our empathy, and thereby our benevolent concern, to other individuals and further into the future, and make these attitudes less discriminatory. We can also extend our empathy to a greater number of people, by voluntarily imagining the suffering of a single, arbitrary individual from a large group and telling ourselves that the suffering of each individual of the group is as real and morally bad as is the suffering of this individual and, thus, that our concern for the sufferings of the entire group should be proportionally greater. This is likely to make our concern for the collective suffering significantly greater than our concern for a single individual’s suffering, though hardly as great as it ideally should be.

In sum, by means of our reason we can counteract the fact that our empathy is spatio-temporally biased, unjustifiably discriminatory, and innumerate, and develop a more reflective empathy, though we would be hard put to overcome completely these shortcomings of our spontaneous empathy. Such a reflective empathy would motivate a correspondingly more reflective and justifiable altruistic concern, as well as greater, more rational prudential concern for our future selves. In other words, we are capable of a sort of motivational bootstrapping: we find ourselves concerned about others because we automatically empathize with them to some degree, and this concern could motivate us to reflect on the unjustifiability of the grounds that tend to eclipse our automatic empathy for these individuals, and modify our concern for them in ways we see as rationally justified by voluntarily imagining more vividly what it is like to be them. By this procedure we could surmount obstacles that evolutionary programming has put in the path of our empathy and benevolent concern. This is clearly a superior strategy to trying to divest ourselves of empathy because it is misdirected or restrictive, and thereby risk divesting ourselves of concern for others.

As already indicated, the point can also be made in terms of prudential concern for our own more distant future. It would be a poor strategy to give up empathizing with our possible suffering in the distant future, say from lung cancer from smoking cigarettes. Rather, we should develop a more reflective empathy which extends further into our future, by heeding our reason’s advice to imagine more distant suffering as vividly as we spontaneously imagine suffering that is closer in time. This strategy is more likely to generate motivation to give up smoking now than refraining from such a voluntary direction of imagination.

## Empathy, Justice and Anger

Some grounds for excluding individuals from, or pushing them to the periphery of, empathy and concern are however warranted. Bloom notes that ‘you feel more empathy for someone who treats you fairly than for someone who cheats you’ [1, p.68]. We put this down to our being equipped with a *sense of justice or fairness*. This sense expresses itself not only in our making *judgments* about what is (un)just or (un)fair, but also in our being *motivated* to some extent to rectify what we judge to be unjust or unfair.

Like many other animals, we practice the *tit-for-tat strategy* which consists in responding in the same coin: being angry at and inclined to punish those who harm us, or those close to us, and being grateful and inclined to reward those who benefit us, or those close to us. From an evolutionary point of view, it is not hard to comprehend why we abide by this strategy. But in contrast to (most) other animals, we have the power to contemplate whether our spontaneous angry or grateful reactions are justifiable. The upshot of this contemplation may be that after all an angry and punitive response would not be just or justifiable because, say, the harm inflicted on us was accidental or unavoidable, or that it was inflicted in order to protect somebody else from much greater harm. So, reason may command us to hold back our spontaneous angry and punitive responses.

We may however fail to comply with this command if we empathize strongly with the victims of the harmful act but not at all with its perpetrator. Indeed, such biased empathy might even get in the way of our making reasonable judgments about what is justifiable. We might be so incensed by the fact that we have been harmed that we overlook that the infliction of the harm was unavoidable or justifiable, just as we might be unable to control our temper and retaliate, though we realize that it is unjustifiable. This is of course less likely to happen if we empathize not only with the victims of a harmful act but also with its agent.

Although Bloom concedes that ‘empathy can serve as the brakes’ on aggression and violence for such reasons, he argues that ‘it’s just as often the *gas*’ [1, p.188]: ‘the empathy one feels toward an individual can fuel anger toward those who are cruel to that individual’ [1, p.208]. But it is not empathy with the cruelly treated victim that *triggers* the aggression toward the offender; it is our adherence to the tit-for-tat strategy that drives us to punish agents who are cruel to those we care about. Certainly, if we empathize with victims – but not with the aggressors – this will *amplify* our aggression, but it is not empathy, or its absence, that *makes* us angry at aggressors. If it is claimed that it is such an amplification of anger beyond reason that is what makes empathy undesirable in this connection, it should be retorted that it offers the compensating good of serving as a brake on our anger if we direct it at the aggressors. Surely, this is not what you expect of something that is properly characterized as ‘a powerful force for war and atrocity’ [1, p.9].

In any case, it is doubtful that the removal of empathy is a recommendable remedy (were it feasible). Although our sense of justice by itself motivates us to do what we judge to be just, the risk is that this motivation will be so weak in many of us that we will do little to rectify instances of injustice. For when we attempt to punish offenders, or extract compensation from them, we usually have to stick our necks out, and we are disinclined to do so for people we do not care about. Likewise, we are also likely to incur costs should we reward those who have acted justly. Benefiting people who are unfairly worse off or in greater need than others when this is simply the result of bad luck and not the wrong-doing of any moral agents is of course also costly. A better strategy than removal of empathy would seem to be to involve empathizing with those at the receiving end of unfair acts and misfortunes, but balancing it with a reasonable amount of empathy for possible offenders.

The importance of empathy as a brake on unjustified aggression and violence may be even clearer in the case of psychopaths. Bloom’s conclusion about them is: ‘They do tend to be low in empathy. But there is no evidence that this lack of empathy is responsible for their bad behavior’ [1, p.201]. Here it is important to bear in mind that empathy has a function to fill not only with respect to others, but with respect to our own future as well. Psychopaths are characteristically unconcerned not only about other individuals, but about their own long-term future (and past), too. Hence, their ‘lack of realistic long-term goals’, their ‘impulsivity’ and ‘irresponsibility’ [1, p.198]; their being ‘relatively indifferent to punishment’ and ‘unmoved by love withdrawal’, as mentioned by Prinz [1, p.218]; cf. [3, p.222]. Now suppose – as seems true – that psychopaths are also prone to aggression and excessive vanity. It is obvious that such people could easily be driven to crime and other immoral behaviour to gain short-term profits because neither concern for others nor fear of punishment and social condemnation of themselves will hold them back.

Like the instinctive grounds for excluding people from empathy and altruistic concern, the instinctive grounds for how justice requires people to be treated are then subject to revision in light of reason. These grounds seem partly to overlap. For instance, earlier on in human history the fact that some people were deformed or disabled appears to have been regarded not only as a ground for excluding them from empathy and concern but, even more absurdly, as grounds making it just (deserving, fitting etc.) that they be worse off than others.

Prinz considers the possibility ‘to overcome the selective nature of empathy by devising a way to make us empathize with a broader range of people’ [2, p.228], but rejects it because he thinks that empathy is ‘intrinsically biased’ [3, p.229]. Instead, he favours a ‘less demanding’ alternative which dispenses with empathy. This alternative appeals, in the negative case, only to feelings of disapprobation like anger and guilt, and types of action that evoke them:

If we focus our moral judgments on *types of actions* (stealing, torture, rape, etc.) and make an effort *not* to reflect on the specific victims, we may be able to achieve a kind of impartiality… *any* focus on the victim of a transgression should be avoided, because of a potential bias. [2, p.228]; [3, p.220]

The emotions of disapprobation are more powerful motivators of moral action than empathy, according to him. And ‘anger can be conditioned through imitation. If we express our outrage at injustice, our children will feel outrage at injustice’ [2, p.229]. Sure, but children can be taught to feel outrage at types of actions that many of us would not nowadays regard as morally wrong, such as working on Sundays or consensual homosexual acts between adults. To explain why such acts are not *morally* wrong while others are, it seems that we would have to refer to the fact that they do not harm or make anyone unjustly worse off, whereas morally wrongful acts do. But then it can be objected, again, that it seems that the realization that some individuals have been unjustly harmed will elicit little, if any, anger or indignation if we are unconcerned about those harmed and that we are likely to be unconcerned about them if we have never empathized with them. Prinz recognizes that we need ‘a keen sense that human suffering is outrageous’ [2, p.229], but can we develop such a sense if we strive for an impartiality that ‘involves bracketing off thoughts about victims’ [2, p.229]? And what about cases in which human suffering is due to bad luck which does not involve any moral wrongdoing, like instances of congenital disabilities and natural disasters? In such cases, emotions like anger, indignation and outrage are out of place or irrational.

Additionally, anger can be as at least biased and immoral as empathy, say, the anger you feel at your opponent because you have lost a game, though you realize that the game was perfectly fair, and you entered into it voluntarily. Prinz is on firmer ground when he speaks about outrage and indignation, since these emotions seem to be anger which you believe to be morally justified. But because of the excessiveness of your self-concern, these emotions can still be overblown when you yourself are the victim of immoral behaviour.

## Whether Empathy Is Necessary for Moral Concern, or could Block it

So much about exploiting empathy for good purposes. It might be objected, however, that in order to act out of concern, we evidently do not need to empathize. For instance, we might grab a pedestrian to prevent him or her from stepping out in front of a bus, without having had time to do any empathizing. True, but this may be because we have acquired a *habit* of acting in such ways because we have often in the past empathized with people in similar circumstances and, thereby, acquired a *standing* motivation to help them out. Thus, empathy may be necessary to make us concerned about others to start with, without subsequently being necessary for action out of concern on each and every occasion.^7^

In the case of the pedestrian, there are no competing interests. But when there is such competition, one party often claims our spontaneous concern at the expense of others. Then our voluntarily directed, reflective empathy should step in to rectify matters. Consequently, the fact that we have developed standing desires that enable us to do the right thing on many occasions does not mean that engaging in empathy has been rendered redundant. Sooner or later spontaneous, selective empathy will assert itself, and the combat against its distortions must be resumed.

Prinz relates studies that show that people who found a dime in a phone booth are much more likely to help a passerby who has dropped some papers than people who did not find a dime. He concludes that a ‘small dose of happiness seems to promote considerable altruism’ [2, p.220]. Certainly, a standing disposition to be of assistance is more likely to manifest itself if we are happy. When we feel that things are going well for ourselves, we are more bent on making efforts to assist those who are less fortunate and imaginatively to put ourselves in their shoes. Prinz thinks that this imaginative act could reduce the inclination to help because the ‘vicarious distress’ he sees empathy as involving ‘presumably has a negative correlation with positive happiness’ [2, p.220]. But here it should be remembered, first, that empathy with the distressed does not involve actually *feeling* distress, but *imagining* feeling the distress others are believed to be feeling, which is less of a negative experience. Secondly, if we can do something to help the distressed, we may end up actually *feeling* satisfied.

Prinz also affirms that guilt ‘is a great motivator’ on the ground that subjects were much more disposed ‘to make some fund-raising calls for a charity organization after they administered shocks to an innocent person’ [2, p.221]. But the fact that we are inclined to make up for an immoral act we believe we have committed by doing good is an expression of our sense of justice, and has been seen this does not rule out being motivated by empathy as well.

There is however something backward about Prinz’ conception of guilt as a motivator. He claims: ‘If we anticipate that an action will make us feel guilty, we will thereby be inclined to avoid the action’ [2, p.219]. However, if an act makes us feel guilty, we must take it to be wrong. Now he also maintains: ‘A person who judges that stealing is wrong, for example, will be motivated to resist the urge to steal’ [2, p.219]. It follows that if we anticipate that an act will make us feel guilty once we have committed it, we already conceive of it as wrong – since our committing it does not *make* it wrong – and, hence, we will already ‘be motivated to resist the urge’ to commit it, independently of the guilt we would feel were we to commit it.

It should be stressed, however, that empathy is not an end in itself; it is emphatically not the case that the more we empathize, the better. Voluntarily empathizing is a means to boost motivation to assist those in need, to enhance concern for them. But, as Bloom points out [1, pp.133–46], people can empathize too much, so much that it can be harmful both to themselves and to those in need of their help. It can ‘burn out’ empathizers, make them depressed and emotionally exhausted. This is not surprising: after all, imagining feeling suffering is an unpleasant experience in itself, though not as unpleasant as actually feeling the suffering imagined. And it is not hard to understand how, say, doctors who are absorbed by acts of imagining the suffering of their patients might be overwhelmed and paralyzed by what they imagine and unable to help their patients effectively.

But conceding that empathy can be excessive, that there can be too much of it, is not conceding that it is redundant, that there cannot be too little of it. In moderate measures it may be an efficient means of enhancing flagging concern. A Buddhist scholar quoted by Bloom states the appropriate recipe thus: ‘meditation-based training enables practitioners to move *quickly* from feeling the distress of others to acting with compassion to alleviate it’ ([1, p.141], emphasis added).^8^ Bloom himself says essentially the same: ‘we know that feeling empathy for another makes you more likely to help them’, though ‘too much empathy can be paralyzing’ [1, pp.155–6]. However, even if we do not get caught up in excessive empathy, but use it wisely to quicken our benevolent concern, it should be observed that we run the risk of being burnt out: for those who are greatly concerned to relieve the suffering in a world so full of misery as this one, awareness of the fact that, even if they were to comply with a very demanding morality, they could at best only relieve a tiny fraction of it could well be crushing. But the fact that a state of mind is crushing does not count against it being the state of a morally enlightened person, at least not as long as this is not allowed to prevent complying with moral demands. In itself, the contentment of a moral agent is not an objective of morality.

It could be desirable to enhance our capacity for empathy and concern by drugs, like oxytocin etc. if the discomfort this would cause us would be outweighed by the greater amount of good we would thereby accomplish. It might be asked whether these drugs work by making us more disposed to empathize, or be more motivated by the outcome of our empathizing. But these aspects are not easily separable, since if we become more disposed to engage in acts of imagination, these acts will be more protracted, and their content more vivid or detailed and, thus, it will have greater motivational impact. However that may be, such enhancement cannot render a reflective direction of imagination superfluous.

## Conclusion: Better to Reform Empathy than Remove it

All in all, the picture that emerges is this. We have beliefs about how other individuals feel and how we can help them to feel better. There is both a set of properties such that: (1) if we believe individuals have any of these properties, this facilitates spontaneous empathy with these individuals, i.e. disposes us to imagine spontaneously how they feel, and (2) a set of properties such that if we believe that individuals have any of them, this hinders spontaneous empathy with them. In the former case, we will be spontaneously concerned about the well-being of these individuals; in the latter case, it will take voluntary reflection to empathize and be concerned about the individuals in question. We are also in possession of a sense of justice or fairness which not only animates us to benefit those whom justice requires to be benefited, but also to harm those whom justice requires be harmed. If, however, we do not empathize with individuals who should be benefited, or have been harmed, we will often not be sufficiently motivated to rectify injustices inflicted on them. Similarly, the incentive to punish is reduced if we empathize with those who have unjustly harmed.

Because we are equipped with a power to scrutinize the rationality of our responses, we might realize that some of the properties that we unthinkingly use to exclude individuals from our spontaneous concern do not in fact justify such exclusion. This would provide us with reason voluntarily to imagine more vividly the feelings of these individuals who have previously received little empathy from us and, as a result, be more concerned about their welfare, though presumably not as much as we are concerned about those with whom we spontaneously emphasize. Still, we should be wary not to employ voluntary empathy excessively, so that it gets in the way of acting out of concern.

This is basically in agreement with a picture we have earlier put forward [5]. We then suggested that there are two ‘core moral dispositions’ [5, p.108], altruism and a sense of justice. They seem to be biologically based in part and, therefore, in principle open to enhancement by biomedical means. But we pointed out that such enhancement is not sufficient because our spontaneous altruistic concern is subject to various distortions, like the bias towards the near future [5, pp.27–8], innumerateness or insensitivity to numbers [5, p.30]), and in-group bias [5, pp.119–20]. None of these considerations stands up to rational scrutiny. This realization is liable to change the pattern of our spontaneous concern, but our sense of justice is in even greater need of reflective refinement. It comprises a disposition to do what we *think* is just or fair. But what *is* just or fair is a topic of heated philosophical controversy, whether it is a matter of getting what we deserve, or something more egalitarian, etc.^9^

Bloom writes that he has ‘been arguing throughout this book that fair and moral and ultimately beneficial policies are best devised without empathy’ [1, p.207]. It might indeed be true that such policies are best *devised* without empathy, but it is doubtful whether we can be best *motivated* to act in accordance with them without empathy. Consider again a simpler situation in the realm of prudence. If we have to devise a general policy for possible future circumstances in which we have to choose between a smaller, but still acute, pain the same day and a significantly bigger pain a week later, it is easy to tell what the best policy is: to opt for the smaller pain the same day. The hard bit is to stick to this policy when the smaller pain will occur later today. To avoid backsliding we then have to counteract our spontaneous empathy with ourselves in the imminent future by voluntarily imagining what it will be like for us to suffer the greater pain a week later. We shall most probably never succeed in voluntarily directing our empathy to the extent that we become temporally neutral and care as much about the more remote as the closer future. But it is still better to have such an imperfectly reformed empathy than being without all empathy, spontaneous as well as reflective.

Bloom seems to concede something like this when, in speculating about how he would genetically engineer a child, he writes that he would be wary of removing empathy, but ‘would ensure that it could be modified, shaped, directed, and overriden by rational deliberation’ [1, p.212]. This would be what we have called reflective empathy. Since he thinks that this child should be equipped with empathy, he must think it can do some good. His case against empathy must then rest on it being impossible in fact to modify it to the extent that its existence is better than its non-existence. Granted, empathy cannot be modified to the extent that it perfectly concords with the deliverances of rational deliberation, but it can be sufficiently reshaped that we are better off with it than without it, since in the latter case it seems that we would risk being totally unmotivated by any mental states beyond those of ourselves in the present.

Bloom concludes about empathy: ‘its negatives outweigh its positives – and there are better alternatives [1, p.241]. The present conclusion is to the contrary that it is the positives that outweigh the negatives, and that there is no better alternative to empathy. Bloom seems to have reason in mind, but reason is not an *alternative* to empathy: it needs empathy as a motivator, and empathy needs reason for its motivational force to be properly directed and encompassing. Compassion and concern on which he places higher value than empathy are no more reliable as moral compasses. They, too, are biased and parochial, underpinned as they are by empathy, and in need of supervision by reason.^10^

## Footnotes

1 Concurring with Adam Smith that, in contrast to ‘pity’ and ‘compassion’, ‘sympathy’ may ‘without much impropriety, be made use of to denote our fellow-feeling with any passion whatever’ p765 [4], we have earlier employed the term ‘sympathetic concern’ (e.g. p109 [5]).

2 Cf. Coplan’s distinction between self-oriented and other-oriented perspective-taking pp9–15 [6].

3 Thus, empathizing is not imagining having any experience, as Bloom’s claim that it is ‘experiencing what they experience’ p3 [1] could suggest. If, say, we imagine seeing what others are seeing from their points of view, this does not qualify as empathy because it is not any *feeling* that is imagined. Additionally, it is odd to talk about empathizing with somebody who is feeling warm or surprised when these feelings are neither positive nor negative.

4 Experimental evidence for this hypothesis is summarized e.g. in Batson [7].

5 This is a reason why it is important not to confuse empathy with emotional contagion: we cannot directly infect ourselves with emotions at will.

6 Prinz claims: ‘Imagination sounds like a kind of mental act that requires effort on the part of the imaginer’ p212 [2]. But, as for instance Hume stressed, if we have regularly experienced one type of event being succeeded by another type of event, experiencing the first is likely to make us automatically imagine experiencing the other, especially if it is pleasant or unpleasant.

7 Here surfaces a difference between concern, on the one hand, and sympathy, pity and compassion, on the other, for although we might say that your act expressed concern for the well-being of the pedestrian, we would scarcely say that you felt sympathy, pity or compassion for the pedestrian. These emotions do seem to involve empathy on each and every occasion.

8 For a survey of the ancient Buddhist tradition of cultivating empathy, see McRae [8]. In this tradition, ‘empathy as imaginative projection’ ‘is assumed to be highly trainable’, ‘vastly under-utilitized’ p124 [8], and also that it ‘will stimulate compassion’ p125 [8]. That is to say, virtually what we argue.

9 Followers of Hume and Smith, like e.g. Kaupinnen [9], who believe that empathy is involved in moral judgment also contend that it can be regulated, but their view is different, and more contentious, than ours in at least two respects. First, we explore the role of empathy in *one* species of moral *motivation*, not its role in the making of moral *judgments*, let alone *all* kinds of moral judgments. In addition, Kaupinnen thinks that for his purpose empathy as regards reactive attitudes like resentment and gratitude is more central than empathy as regards concern for the well-being of others. Secondly, these theorists propose regulation by reference to an *ideal perspective*, which goes beyond the regulation we have here considered.

10 Many thanks to the reviewers and editor for valuable comments on an earlier version of this paper.
